# Are young people with primary social anxiety disorder less likely to recover following generic CBT compared to young people with other primary anxiety disorders? A systematic review and meta-analysis

**DOI:** 10.1017/S135246582000079X

**Published:** 2021-05

**Authors:** Rachel Evans, David M. Clark, Eleanor Leigh

**Affiliations:** 1 Department of Psychology, IoPPN, King’s College London, London, UK; 2 Department of Experimental Psychology, University of Oxford, Oxford, UK

**Keywords:** adolescents, anxiety, children, cognitive behavioural therapy, social anxiety disorder

## Abstract

**Background:**

Social anxiety disorder (SoAD) in youth is often treated with a generic form of cognitive behavioural therapy (CBT). Some studies have suggested that primary SoAD is associated with lower recovery rates following generic CBT compared with other anxiety disorders.

**Aims:**

This systematic review and meta-analysis investigated recovery rates following generic CBT for youth with primary SoAD *versus* other primary anxiety disorders.

**Method:**

Five databases (PsycINFO, Web of Science, PubMed, Embase, Medline) were searched for randomised controlled trials of generic CBT for child and/or adolescent anxiety.

**Results:**

Ten trials met criteria for inclusion in the systematic review, six of which presented sufficient data for inclusion in the meta-analysis. Sixty-seven did not report data on recovery rates relative to primary diagnosis. While most individual studies included in the systematic review were not sufficiently powered to detect a difference in recovery rates between diagnoses, there was a pattern of lower recovery rates for youth with primary SoAD. Across the trials included in the meta-analysis, the post-CBT recovery rate from primary SoAD (35%) was significantly lower than the recovery rate from other primary anxiety disorders (54%).

**Conclusions:**

Recovery from primary SoAD is significantly less likely than recovery from any other primary anxiety disorder following generic CBT in youth. This suggests a need for research to enhance the efficacy of CBT for youth SoAD.

## Introduction

Social anxiety disorder (SoAD) is characterised by an intense fear of embarrassment or negative evaluation by others which causes significant distress and functional impairment. It is common, with a lifetime prevalence of 12% (Kessler *et al*., 2005). It has a median age of onset of 13 years (Kessler *et al*., 2005) and a stable, chronic presentation in both young people and into adulthood (Bruce *et al*., 2005). In young people, SoAD is associated with poor academic achievement (Van Ameringen *et al*., 2003) and peer victimization (Ranta *et al*., 2009). SoAD continues to be associated with functional impairments across work and social domains in adulthood (Aderka *et al*., 2012). Moreover, adolescent SoAD is associated with development of subsequent depression (Stein *et al*., 2001), which itself is predictive of a range of a range of functional impairments (McKnight and Kashdan, 2009).

For the treatment of SoAD in adults, the National Institute of Health and Care Excellence (NICE, 2013) recommend individual cognitive behavioural therapy (CBT) based on a disorder-specific model (i.e. Clark and Wells, 1995; Rapee and Heimberg, 1997). It is unusual for psychological interventions to show superior outcomes to other active treatment conditions. However, CBT for SoAD following the Clark and Wells model has been shown to be more effective than exposure therapy (Clark *et al*., 2006), group CBT (Mörtberg *et al*., 2007; Stangier *et al*., 2003) interpersonal psychotherapy (Stangier *et al*., 2011), psychodynamic psychotherapy (Leichsenring *et al*., 2013) and medication (Clark *et al*., 2003; Mörtberg *et al*., 2007; Nordahl *et al*., 2016; Yoshinaga *et al*., 2016).

A recent review identified empirical studies supporting the applicability of the Clark and Wells (1995) model to adolescent social anxiety (Leigh and Clark, 2018) and there is emerging evidence of the effectiveness of cognitive therapy for adolescent SoAD based on this model (Ingul *et al*., 2014; Leigh and Clark, 2016). This approach includes an individualised formulation based on a disorder-specific model of SoAD (e.g. Clark and Wells, 1995) followed by a range of techniques aimed at targeting maintenance factors identified in this model, for example using tailored behavioural experiments, attention training and memory rescripting. In contrast, generic CBT typically involves components such as psycho-education, graded exposure, problem solving and coping strategies which are not included in cognitive therapy (Spence *et al*., 2017). However, generally there has been a lack of research into disorder-specific interventions for child and adolescent SoAD, as reflected in the NICE guidelines which do not specifically recommend disorder-specific CBT for SoAD in youth (NICE, 2013). Indeed, as highlighted by Creswell *et al*. (2014), there has historically been a lack of research investigating disorder-specific models and therefore treatments across the range of anxiety disorders in youth. In a recent review, Creswell *et al*. (2020) have emphasised the need to move away from the historical ‘one-size-fits-all’ approach to disorder-specific CBT for child and adolescent anxiety disorders.

The efficacy of generic CBT for the treatment of anxiety disorders in young people has been supported by several reviews, with odds ratio for recovery of 3.3–7.85 following CBT compared with waitlist control (Cartwright-Hatton *et al*., 2004; James *et al*., 2013). However, several authors have suggested that youth with SoAD may experience poorer treatment outcomes than those with other anxiety disorders. In an analysis of predictors of outcome from a large randomised controlled trial (RCT), Ginsburg *et al*. (2011) found that young people with SoAD anywhere in their diagnostic profile were less likely to be in remission following CBT, compared with those without SoAD: remission was observed in only 41% of those with SoAD, compared with 72% of those without. Hudson, Keers *et al*. (2015) integrated data from 1519 children who received a course of CBT for anxiety in RCTs across 11 international sites, to reveal that youth with primary or secondary SoAD experienced significantly lower rates of recovery from their primary diagnoses following CBT, compared with those without SoAD. A similar pattern has been found at long-term follow-up: Kerns *et al*. (2013) reported that presence of SoAD anywhere in the profile was predictive of poorer diagnostic outcomes 7.4 years post-CBT as delivered in an RCT.

Whilst some individual studies have demonstrated poorer outcomes for youth with SoAD compared with those with other anxiety disorders, findings to date from reviews have been mixed. In a review investigating pre-treatment predictors of outcome following psychological treatments for child and adolescent anxiety, Knight *et al*. (2014) concluded ‘emerging evidence’ to suggest that primary SoAD predicted poorer outcome, whereas generalised anxiety disorder predicted superior outcome. However, in a review of child and family characteristics as predictors of outcome from cognitive therapy, Lundkvist-Houndoumadi *et al*. (2014) concluded there was insufficient evidence to suggest that any primary diagnosis was predictive of treatment outcome. More recently, Walczak *et al*. (2018) conducted a review investigating whether co-morbidity predicted outcomes following CBT for child anxiety disorders. This review concluded that SoAD may be associated with poorer outcomes following CBT, although it did not specifically focus on recovery outcomes for those with primary SoAD and did not report a quantitative synthesis (meta-analysis of results). Therefore, whilst this provides further evidence to suggest a relationship between SoAD and recovery rates, it does not provide a clear answer regarding outcomes for young people with primary SoAD compared with other anxiety disorders.

Given the prevalence of SoAD and associated functional impairments in young people, and suggestions from several authors that SoAD may be less responsive to generic CBT than other anxiety disorders, this issue clearly merits further investigation. Although previous reviews have attempted to identify factors which predict outcomes from CBT, the current review is the first to specifically investigate the role of primary SoAD in recovery from generic CBT. If it is the case that young people with primary SoAD experience significantly poorer outcomes following generic CBT, this would necessitate further research into disorder-specific interventions for social anxiety in young people. Such a finding would also have implications for clinical services, as it would suggest that young people with primary SoAD may require alternative treatment such as disorder-specific CBT (e.g. Ingul *et al*., 2014; Leigh and Clark, 2016).

The efficacy of disorder-specific CBT for SoAD, generalised anxiety disorder (GAD), separation anxiety disorder (SAD) and specific phobia (SP) has recently been considered in a systematic review conducted by Oldham-Cooper and Loades (2017). This review focused on one generic format of CBT (individual CBT following the ‘Coping Cat’ manual; Kendall, 1994) compared with disorder-specific psychological treatments. The review concluded that there was no evidence to favour disorder-specific over generic CBT for anxiety disorders. However, Oldham-Cooper and Loades only included one trial of disorder-specific CBT for SoAD, clearly limiting the strength of findings related to this diagnosis. A recent comparison of online generic *versus* disorder-specific CBT for SoAD (Spence *et al*., 2017) found that whilst the disorder-specific intervention was associated with greater reductions in SoAD maintenance processes and improvements in functioning, there was no significant difference in recovery outcomes at 12 weeks or at 6 months follow-up. However, the sample size of this trial did not provide sufficient statistical power to detect a difference between these two active treatment conditions. Moreover, it is important to clarify that a lack of evidence of the superiority of disorder-specific CBT does not necessarily demonstrate equivalence of outcomes for young people with SoAD compared with other anxiety disorders. It is quite possible for it to be true that current disorder-specific models of CBT have not demonstrated superiority over generic treatments for SoAD, and that young people with SoAD have poorer outcomes from generic CBT.

It is clear from the discussed literature that SoAD is a common difficulty in young people with disabling implications. The suggestion from several trials that young people with SoAD experience poorer outcomes from CBT, coupled with the lack of overall clarity from reviews of the topic, necessitate further investigation. Better understanding of this issue is crucial, as this can inform the development and implementation of better psychological therapies for this common disorder in young people. The present review therefore set out to investigate diagnostic outcomes following generic CBT for young people with primary SoAD in comparison with those with other primary anxiety disorders. Specifically, we set out to investigate remission from primary anxiety diagnosis (defined as no longer reporting symptoms in the clinical range on either young person or parent report on diagnostic assessment tools), at post-treatment, in children and/or adolescents with an average age of 7–18 years. In contrast to the Oldham-Cooper and Loades (2017) review, the present review included any form of CBT (i.e. following a range of manualised approaches and group/individual formats). This approach was taken in order to maximise generalisability of conclusions, both in terms of numbers of trials to be included and to reflect the varied ways in which CBT is delivered to young people with SoAD in routine clinical settings. The present study set out to complete a meta-analysis as well as systematic review, to overcome the common issue of insufficient power within studies to compare outcomes for participants with different primary anxiety diagnoses.

## Method

### Protocol and registration

The protocol for the review is available online at: https://www.crd.york.ac.uk (ID: CRD42019122593). The review followed PRISMA (2009) guidelines (Moher *et al*., 2009). The PRISMA checklist can be found in Appendix A in Supplementary material.

### Eligibility criteria

Inclusion and exclusion criteria were pre-determined and are available at: https://www.crd.york.ac.uk (ID: CRD42019122593). The inclusion criteria were: (i) participants aged 7–18 years, or mean age of 8–17 years, (ii) RCTs of any type of CBT for anxiety disorders (e.g. group/individual format, with/without parental involvement), (iii) includes validated diagnostic assessment at pre- and post-intervention (e.g. ADIS-IV-C/P; Silverman and Albano, 1996), (iv) includes participants with primary SoAD and other anxiety disorder(s) [generalised anxiety disorder, separation anxiety disorder, panic disorder (PD) with/without agoraphobia (AP), AP without PD, SP, anxiety disorder not otherwise specified (ADNOS)], (v) reports recovery outcomes separately for young people with each of the included primary anxiety diagnoses, or an analysis of the moderating effect of primary social anxiety on outcome, (vi) published in a peer-reviewed journal, (vii), written in English, and (viii) published 1990–2019. The exclusion criteria were: (i) a focus on anxiety in participants with diagnosed autism spectrum disorder (ASD), attention deficit hyperactivity disorder, or intellectual disabilities, (ii) sample size <10, (iii) reports replicated data only, and (iv) in line with Oldham-Cooper and Loades (2017), young people with a diagnosis of obsessive compulsive disorder (OCD), post-traumatic stress disorder (PTSD) or selective mutism were excluded. OCD and PTSD were excluded because they are not defined as anxiety disorders within *DSM-5*. Young people with a diagnosis of selective mutism were excluded because standard CBT approaches have required adaptation for this group (Hudson *et al*., 2001).

### Information sources

Five databases (PsycINFO, Web of Science, PubMed, Embase, Medline) were searched for articles within the date range of 1990–2019. Hand forward and back searching was conducted by reviewing the reference lists of existing relevant reviews (Cartwright-Hatton *et al*., 2004; James *et al*., 2013; Knight *et al*., 2014; Lundkvist-Houndoumadi *et al*., 2014; Oldham-Cooper and Loades, 2017; Reynolds *et al*., 2012).

### Search

PsycINFO, Web of Science and PubMed were searched on 21 January 2019, and Embase and Medline were searched on 11 March 2019. The search of all five databases was re-run on 21 October 2019, to account for any further relevant studies published during the main data extraction phase. The search terms were designed to retrieve all research studies that had evaluated CBT for anxiety disorders in children and adolescents. These were searched in abstracts and titles of all five databases with limits regarding year range (1990–current), language (English) and source (journal articles only). Although studies focusing on people with ASD or intellectual disabilities were excluded from the current review, the research team opted to manually remove these studies rather than state these in the search terms. This approach was used to minimise the risk of the electronic search erroneously excluding papers which met the inclusion criteria. For an example full electronic search, see Appendix B in Supplementary material. The search terms used are outlined below:

*Youth OR adolescent OR adolescence OR Child OR CAMHS OR Teenage OR CAMS OR Young people OR Pediatric OR Paediatric*

*AND*

*Anxiety OR Anxiety disorder OR social anxiety disorder OR social phobia OR social anxiety OR Panic disorder OR Specific phobia OR Agoraphobia OR Separation anxiety disorder OR Generalised anxiety disorder OR Generalized anxiety disorder*

*AND*

*Cognitive behavioural therapy OR Cognitive behavioral therapy OR Cognitive behaviour therapy OR Cognitive behavior therapy OR Cognitive therapy OR CBT*

### Study selection

Studies were initially screened based on title, and studies that clearly did not meet the inclusion criteria were excluded. This process was completed cautiously, and any studies which may have met inclusion criteria were reviewed further based on abstract at the next stage. Examples of papers that could clearly be excluded at title stage were those focused on anxiety in people with diagnosed ASD, those evaluating psychological therapies for anxiety disorders in adults only, and single case studies or other clearly non-RCT designs. Abstracts of remaining studies were then reviewed against the inclusion criteria. Again, if it was unclear from the abstract whether the study met inclusion criteria, it was carried forward to the full text review stage. The final stage was to review the full texts of all studies included based on abstract. The screening and selection process was completed by one researcher (R.E.) and in cases of ambiguity regarding whether studies met inclusion criteria, this was discussed and agreed with a second researcher (E.L.). Studies were included in the meta-analytic synthesis if they provided data on the number of participants who recovered from primary social anxiety and from other primary anxiety diagnoses.

### Data collection process

Data were extracted from each of the papers by two independent researchers (R.E. and M.T.). Prior to data extraction, the research team agreed on which data would be sought for extraction. A spreadsheet was developed by the research team to enable the standardised collection of all relevant data from each paper where available. For trials in which the manuscript did not provide sufficient data for inclusion in meta-analytic synthesis (*n* = 8), authors were contacted to request this information. The required data for inclusion in meta-analysis was provided by four of these eight authors.

### Data extraction variables

Data on the following variables were extracted: participant demographics (age, gender, ethnicity, inclusion/exclusion criteria, number allocated to each condition), intervention characteristics (CBT manual, duration and format of CBT and control group, drop-out rates) and diagnostic outcomes following CBT (diagnostic tool used, timing of assessments, number or percentage of participants who recovered from each anxiety disorder, and any analysis/reporting of relationship between primary diagnosis and recovery rates). All diagnostic outcome data were collected at the first assessment following completion of CBT only.

### Risk of bias in individual studies

Risk of bias in individual studies was assessed using the Cochrane Collaboration Risk of Bias Tool (Higgins *et al*., 2011). Each study was assessed against each of the six criteria of the tool by two independent researchers (R.E. and G.S.). The researchers then met to discuss ratings. Agreement on ratings was high, with the two researchers agreeing on 96% of ratings prior to discussion. The remaining ratings where there were discrepancies between the researchers were resolved through discussion between the researchers (R.E., G.S. and E.L.).

### Data analysis (meta-analysis)

Analyses for quantitative synthesis were conducted using RevMan software (version 5.3). The data included in this analysis were the number of participants who recovered from primary SoAD, the total number with primary SoAD, and equivalent numbers for all other primary anxiety disorders pooled. In the six trials included in the meta-analysis, the single most severe and impairing diagnosis based on youth or parent report was assigned as the primary diagnosis in accordance with ADIS-IV-C/P guidelines. Recovery from primary diagnosis is defined as being below ADIS-IV-C/P clinical severity thresholds for primary diagnosis based on both youth and parent report at post-treatment. A fixed effects model was used, as heterogeneity was acceptable (*I* ^2^ = 0%). Recovery outcomes (number recovered from primary SoAD *versus* other primary anxiety disorders) were compared using odds ratio.

### Risk of bias across studies (meta-analysis)

Risk of bias across studies (publication bias) was examined using a funnel plot.

## Results

### Study selection

**Figure 1.**
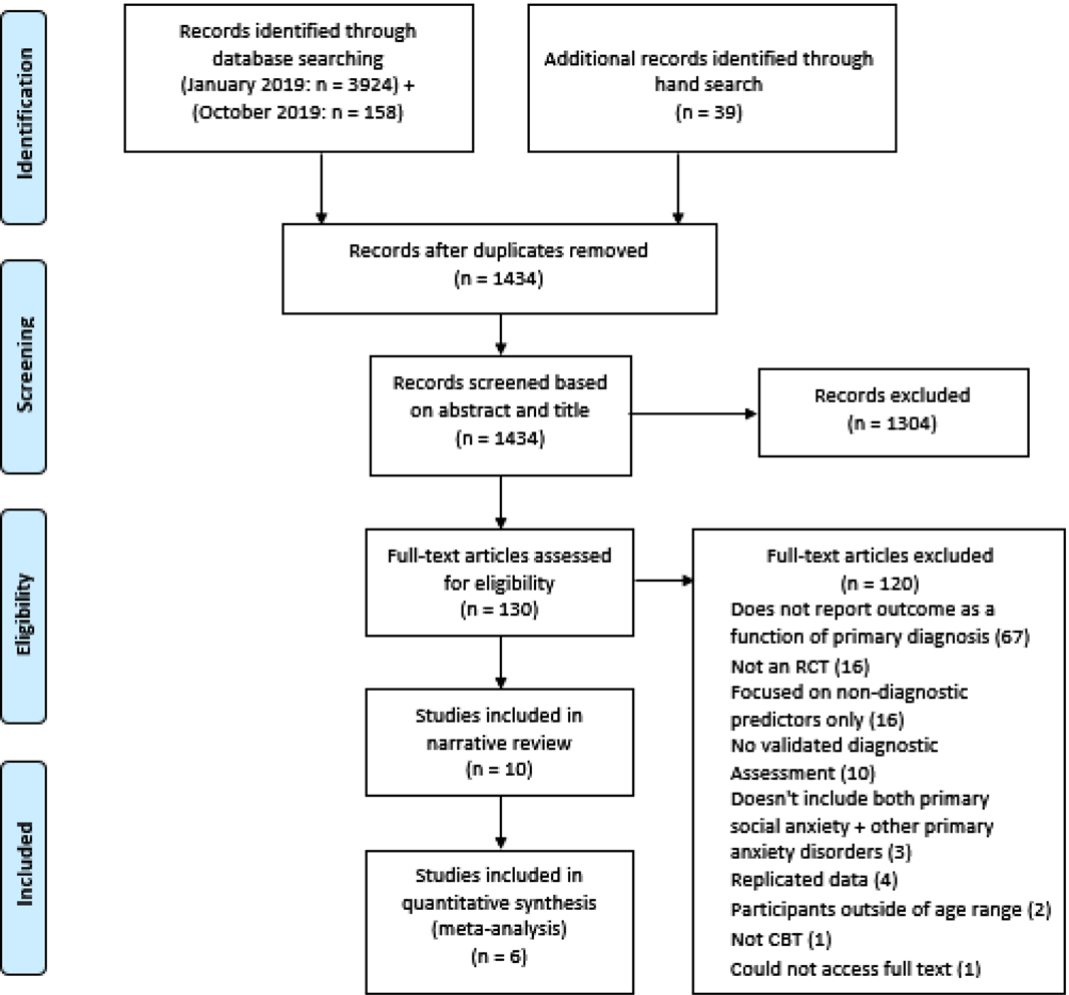
PRISMA flow diagram.

After 956 titles were removed based on title, this left 479 to review based on abstract. At the full text stage, 130 texts were reviewed. Ten texts met criteria for inclusion in the review. Six papers provided sufficient data for inclusion in the meta-analytic synthesis, either in the original paper (*n* = 2) or in response to requests made to the author (*n* = 4). These stages, and reasons for exclusion, are outlined in Fig. 1.

### Study characteristics

Ten published articles were included in the review. These RCTs evaluated a range of formats of CBT, including three of individual CBT (Barrett *et al*., 1996; Silk *et al*., 2018; Suveg *et al*., 2018), two of group CBT (Arendt *et al*., 2016; Shortt *et al*., 2001), two of individual or group CBT (Villabo *et al*., 2018; Wergeland *et al*., 2014; Wergeland *et al*., 2016), two of parent-delivered CBT (Creswell *et al*., 2017; Thirlwall *et al*., 2013; Thirlwall *et al*., 2017), and one of internet-delivered CBT (Stjerneklar *et al*., 2019). Sessions varied in length from 20 min telephone calls (+30 min online sessions) in Stjerneklar *et al*. (2019) to 2 hours (Arendt *et al*., 2016), with an average session length of 59 min (*SD* = 33.71) across the seven studies which reported this information. Children and adolescents receiving CBT ranged in age from 5 to 17 years of age (mean = 10.50, *SD* = 1.78). Further details of the trials are shown in Table 1.

**Table 1.** Characteristics of full texts included in review

Author/year	Country	Total sample size	Age range	% female	Primary anxiety diagnoses included	CBT condition(s)	Diagnostic outcome measure
Barrett *et al*. (1996)	Australia	79	7–14	43%	SoAD, SAD, OAD	12 sessions of individual CBT (Coping Cat; Kendall, 1994)	Anxiety Disorders Interview Schedule for DSM-III (ADIS-III; Silverman and Nelles, 1988)
Shortt *et al*. (2001)	Australia	71	6–10	59%	SoAD, SAD, GAD	12 sessions of group CBT (Friends programme; Barrett, Lowry-Webster & Turner, 2000a-f)	Diagnostic Interview Schedule for Child, Adolescents and Parents (DISCAP; Holland and Dadds, 1995)
Thirlwall *et al*. (2013, 2017)*	UK	194	7–12	48%	SoAD, SAD, GAD, SP, PD without AP, PD with AP, AP	4–8 sessions of individual guided parent-delivered CBT (Willetts *et al.*, 2016)	ADIS-IV-C/P
Wergeland *et al*. (2014, 2016)*	Norway	182	8–15	53%	SoAD, SAD, GAD	10 sessions of individual or group CBT (Friends programme; Barrett, 2004)	ADIS-IV-C/P
Arendt *et al*. (2016)*	Denmark	109	7–16	57%	SoAD, SAD, GAD, SP, PD with AP, AP	10 sessions of group CBT (Cool Kids; Rapee *et al*., 2006)	ADIS-IV-C/P
Creswell *et al*. (2017)*	UK	136	5–12	53%	SoAD, SAD, GAD, SP, PD without AP, PD with AP, ADNOS	8 sessions of individual guided parent-delivered CBT (Willetts *et al*., 2016)	ADIS-IV-C/P
Villabo *et al*. (2018)*	Norway	165	7–13	46%	SoAD, SAD, GAD	14 sessions of individual or group CBT (Coping Cat; Kendall and Martinsen, 2008; Kendall *et al.*, 2006)	ADIS-IV-C/P
Suveg *et al*. (2018)	USA	92	7–12	42%	SoAD, SAD, GAD	10 sessions of individual CBT [Coping Cat; Kendall, 1994; or Suveg and Kendall (unpublished manual)]	ADIS-IV-C/P
Silk *et al*. (2018)	USA	133	9–14	56%	SoAD, SAD, GAD	16 sessions of individual CBT (Coping Cat; Kendall and Hedtke, 2006a,b)	ADIS-IV-C/P
Stjerneklar *et al*. (2019)*	Denmark	70	13–17	79%	SoAD, SAD, GAD, SP, PD without AP, PD with AP, AP,	14 weeks of weekly therapist phone calls + internet-based CBT (ICBT; Lyneham *et al*., 2014)	ADIS-IV-C/P

SoAD, social anxiety disorder; SAD, separation anxiety disorder; GAD, generalised anxiety disorder; OAD, over-anxious disorder; SP, specific phobia; PD without AP, panic disorder without agoraphobia; PD with AP, panic disorder with agoraphobia; AP, agoraphobia without panic disorder; ADNOS, anxiety disorder not otherwise specified. *Included in meta-analysis.

### Risk of bias within studies

**Figure 2.**
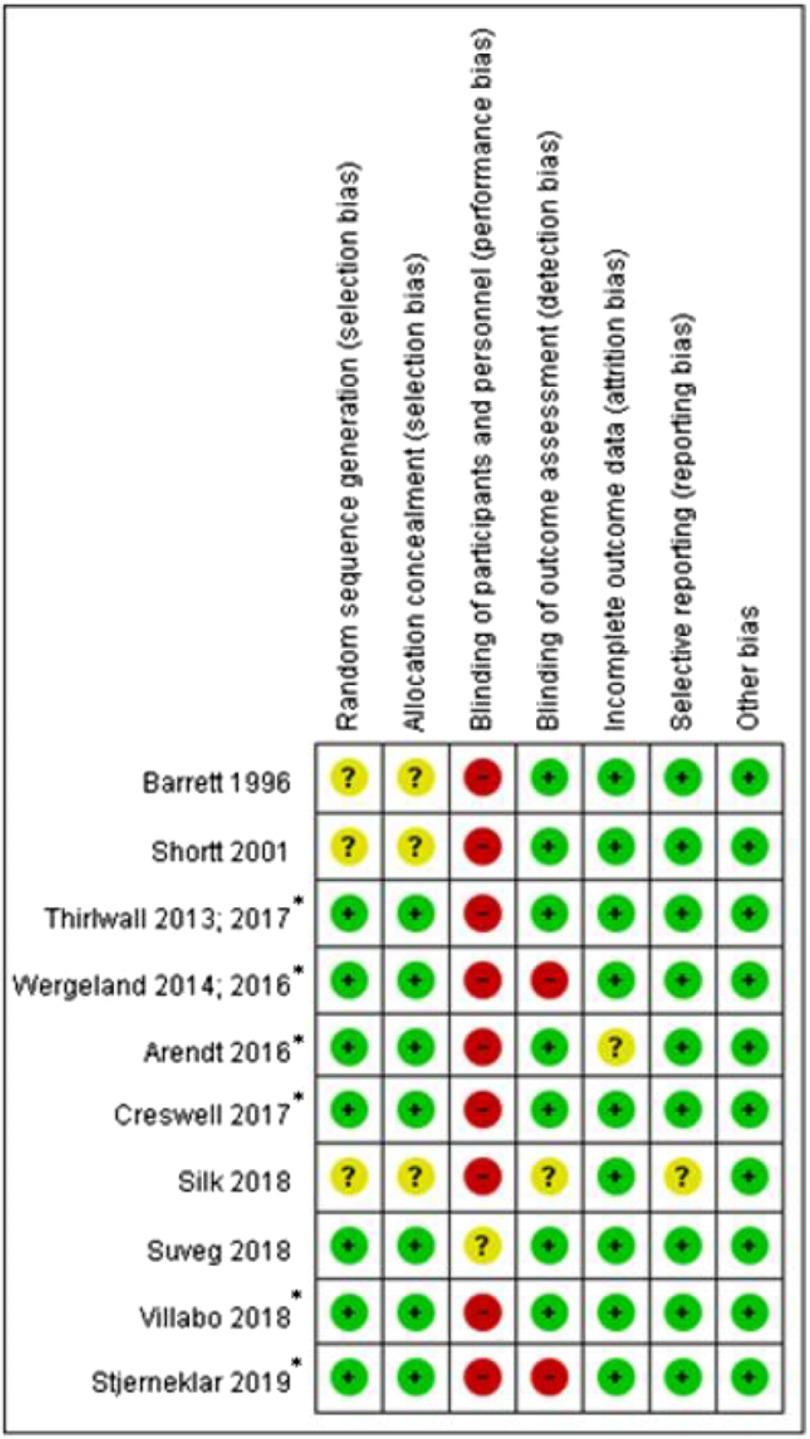
Risk of bias summary for each paper, according to Higgins *et al*. (2011) criteria. *Included in meta-analysis.

Investigation into risk of bias within studies revealed that across five of the six domains, the majority of trials showed low risk of bias. However, all but one trial had a high risk of bias related to participant blinding to treatment condition. This is a common feature of evaluations of CBT, as when comparing CBT with a control condition (e.g. wait list or treatment as usual), it is not possible to prevent participants from knowing which condition they are in. Indeed, it has been suggested in a previous review that this criterion should not be assessed for psychotherapy trials, due to the nature of their design (Reynolds *et al*., 2012). Additionally, two trials (Stjerneklar *et al*., 2019; Wergeland *et al*., 2014; Wergeland *et al*., 2016) had a high risk of bias in outcome assessment, as assessors were not blind to treatment condition at post-treatment. A summary of the risks of bias assessed in the papers is shown in Fig. 2.

### Management of missing data

In eight of the trials, authors reported results based on intention-to-treat principles (with pre-treatment data carried forward in cases of missing data at post-treatment). Two trials (Shortt *et al*., 2001; Thirlwall *et al*., 2013; Thirlwall *et al*., 2017) reported sensitivity analysis which revealed no significant difference in outcomes when missing data were excluded compared with intention-to-treat analysis, and so opted to report results only for young people with available data at post-treatment.

### Narrative review of individual studies

The trials used a range of formats and statistical approaches to present results related to recovery following CBT for young people with different primary anxiety diagnoses. As shown in Table 2, the proportion of young people who recovered from primary SoAD was lower than the proportion recovered from other primary anxiety disorders in seven of the eight trials which presented these data. The only trial which found greater recovery rates for youth with primary SoAD (Creswell *et al*., 2017) was based on a very small sample (*n* = 6) with this primary diagnosis. Furthermore, all results from individual studies regarding the proportion of young people who recovered must be interpreted with caution as the individual studies did not have sufficient statistical power to test the significance of this difference.

**Table 2.** Post-CBT diagnostic outcomes reported in the included texts

Author/year	Sample size on whom CBT recovery outcomes are based^1^	% recovered from primary SoAD post-CBT	% recovered from other primary anxiety disorders post-CBT	Authors’ conclusions regarding association between primary diagnosis and likelihood of recovery
Barrett *et al*. (1996)	53	62%	73%	No significant association between primary diagnosis and recovery rates^3^
Shortt *et al*. (2001)	48	56%	72%	No significant association between primary diagnosis and recovery rates^3^
Wergeland *et al*. (2014, 2016)*	179	27%	42%	A primary SoAD or SAD diagnosis, compared with primary GAD, significantly reduced the odds of recovery post-CBT
Arendt *et al*. (2016)*	101	29%	65%	Participants with primary SoAD were significantly less likely to be recovered at post-treatment than those with any other primary anxiety diagnosis
Creswell *et al*. (2017)*	62^2^	50%	45%	Significant association between primary GAD and post-treatment severity of primary diagnosis (reflecting greater improvement in severity of primary GAD compared with other primary anxiety diagnoses)
Villabo *et al*. (2018)*	165	55%	63%	Not reported
Suveg *et al*. (2018)	92	Not reported	Not reported	No significant difference in recovery rates by primary diagnoses, although co-morbid primary diagnoses were used, and co-morbidity was high (>90%)3
Silk *et al*. (2018)	90	Not reported	Not reported	Pre-treatment diagnosis did not significantly predict or moderate recovery post-CBT^3^
Thirlwall *et al*. (2013, 2017)*	96	38%	49%	Participants with primary GAD were significantly more likely to have recovered from their primary diagnosis at post-treatment than those with all other primary anxiety disorders
Stjerneklar *et al*. (2019)*	32	21%	50%	Not reported

*Included in meta-analysis. ^1^Sample sizes listed here refer only to young people who met diagnostic criteria for primary anxiety diagnoses included in this review. ^2^Participants with sub-clinical primary diagnoses (*n* = 4) excluded. ^3^Statistical power to detect a between-group effect not reported.

Eight trials reported additional analyses of the association between primary diagnosis and recovery post-CBT. Of these, four (Barrett *et al*., 1996; Shortt *et al*., 2001; Silk *et al*., 2018; Suveg *et al*., 2018) reported no significant association between primary diagnosis and recovery rates following CBT. However, none of these trials reported whether there was adequate statistical power to detect a significant difference.

Two trials (Thirlwall *et al*., 2013; Thirlwall *et al*., 2017; Wergeland *et al*., 2014; Wergeland *et al*., 2016) concluded that a diagnosis of primary GAD was associated with significantly higher rates of recovery at post-treatment compared with other primary anxiety disorders (including SoAD). Additionally, Creswell *et al*. (2017) reported greater change in severity of primary diagnosis at post-treatment for young people with primary GAD compared with other primary diagnoses. One trial (Arendt *et al*., 2016) concluded that recovery from primary diagnosis was significantly less likely for participants with primary SoAD compared with all other primary anxiety diagnoses. Arendt and colleagues also identified that youth with primary SoAD were more likely be older than youth with GAD or SAD and were more likely to report a co-morbid mood disorder compared with youth with any other primary anxiety diagnosis. However, the significant association between primary SoAD and lower recovery rates post-CBT remained significant after controlling for age and mood. Further information on these findings is shown in Table 2.

### Quantitative synthesis: meta-analysis

**Figure 3.**
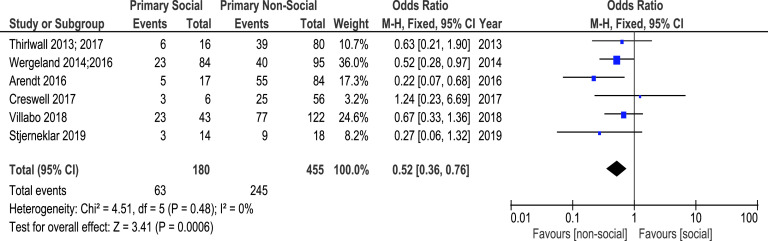
Forest plot showing recovery from primary SoAD ^*vs*^ non-social primary anxiety disorders. Data reported for Villabo *et al*. (2018) are averaged across individual and group CBT conditions.

In order to further examine the association between primary diagnosis and recovery following CBT, a meta-analysis was conducted on the number of young people who recovered from primary SoAD *versus* other primary anxiety disorders at post-CBT, relative to the number who had these diagnoses at pre-CBT. These data were available for six of the 10 studies included in the systematic review (Arendt *et al*., 2016; Creswell *et al*., 2017; Stjerneklar *et al*., 2019; Thirlwall *et al*., 2013; Thirlwall *et al*., 2017; Villabo *et al*., 2018; Wergeland *et al*., 2014; Wergeland *et al*., 2016). These papers presented data on 635 children and adolescents allocated to CBT conditions. Of these, 180 had primary SoAD and 455 had other primary anxiety disorders (SAD = 195, GAD = 180, SP = 56, PD without AP = 6, PD with AP = 4, AP = 12, ADNOS = 2). As heterogeneity was low (*I*² = 0%), a fixed-effects model was used. The meta-analysis revealed that the likelihood of recovery from primary diagnosis was significantly lower for participants with primary SoAD than those with other primary anxiety disorders, OR = .52 (95% CI: .36, .76), *z* = 3.41, *p*<.001. These results are shown in Fig. 3. As a sensitivity analysis, this meta-analysis was repeated using a random-effects model. This revealed a highly similar pattern of results to the fixed-effects model.

### Risk of bias across studies: meta-analysis

A funnel plot was examined of the six studies included in the meta-analysis. This did not indicate evidence of publication bias (see Appendix C in Supplementary material).

## Discussion

This review investigated the question of whether young people with primary SoAD were less likely to recover following generic CBT compared with those with other primary anxiety disorders. In the systematic review, the proportion of young people who recovered from primary SoAD was lower than the proportion who recovered from any other primary anxiety disorders in seven of the eight trials which presented these data. However, these trials did not individually have sufficient statistical power to test for the significance of these differences. In terms of the trial authors’ own analysis of the relationship between primary diagnosis and recovery, four trials (Barrett *et al*., 1996; Shortt *et al*., 2001; Silk *et al*., 2018; Suveg *et al*., 2018) reported no significant association. However, again this must be understood in the context that these trials individually did not have sufficient statistical power to detect such a difference. Nonetheless, three trials concluded that primary GAD was associated with superior diagnostic outcomes post-CBT, and one trial specifically concluded that primary SoAD was associated with poorer recovery rates following CBT compared with other anxiety disorders.

The results from the quantitative synthesis were clearer. When combining the results of six trials which presented sufficient data for inclusion (Arendt *et al*., 2016; Creswell *et al*., 2017; Stjerneklar *et al*., 2019; Thirlwall *et al*., 2013; Thirlwall *et al*., 2017; Villabo *et al*., 2018; Wergeland *et al*., 2014; Wergeland *et al*., 2016), this revealed that 35% of young people with primary social anxiety recovered from this diagnosis following generic CBT, compared with 54% of young people with other primary anxiety disorders. The odds ratio statistic revealed that young people with primary SoAD were 48% less likely to have recovered from this diagnosis post-CBT compared with those with any other primary anxiety disorder. This difference was highly statistically significant. These results complement previous findings that social anxiety anywhere in the diagnostic profile is associated with poorer outcomes following generic CBT (Ginsburg *et al*., 2011; Hudson, Keers *et al*., 2015; Kerns *et al*. 2013). This has also been suggested in a recent review investigating the role of co-morbidity in outcome following CBT (Walczak *et al*., 2018). The present review is the first to demonstrate that primary social anxiety specifically is associated with lower recovery rates following CBT.

This review also builds on the conclusion of the 2014 review by Knight and colleagues (2014) which found emerging evidence to suggest that primary SoAD was predictive of poorer outcomes following CBT. However, these results contrast with the conclusion of Lundkvist-Houndoumadi *et al*. (2014) that there was no significant evidence to suggest that any primary diagnosis was predictive of outcome following CBT for youth anxiety disorders. This is likely due to the availability of trials at the time of Lundkvist-Houndoumadi and colleagues’ review, as eight of the trials in the current review (including all six included in the meta-analysis) were published after Lundkvist-Houndoumadi and colleagues’ search was conducted. This may reflect changing trends in the reporting of data: amongst trials which met all other criteria for inclusion in this review, only 7% published prior to 2014 reported an analysis of the relationship between primary diagnosis and recovery, compared with 32% in those published from 2014 onwards. Furthermore, it highlights the importance of reporting results as a function of primary anxiety diagnosis in trials of CBT for child and adolescent anxiety disorders.

These results have clear clinical and research implications. The finding that only 35% of young people with primary social anxiety recovered following generic CBT suggests that treating primary SoAD with current generic models of CBT is unlikely to enable recovery in the majority of cases. However, there is a lack of evidence to suggest what approach may improve CBT outcomes for this population. There is evidence of the effectiveness of social skills interventions for SoAD in pre-adolescent children (Beidel *et al*., 2000; Beidel *et al*., 2005). For the treatment of SoAD in adolescents, a recent case series presented promising results using an adapted version of the Clark and Wells (1995) adult disorder-specific model of CBT (Leigh and Clark, 2016). Additionally, an RCT has reported that adolescents with SoAD experienced significantly greater symptom reduction following CBT based on the Clark and Wells (1995) model compared with a generic CBT approach (Ingul *et al*., 2014). However, a review completed by Oldham-Cooper and Loades (2017) concluded that there was not sufficient evidence that existing disorder-specific CBT was superior to generic CBT for SoAD in youth. In light of the current review’s findings, it is important that disorder-specific interventions such as those evaluated by Leigh and Clark (2016) and Ingul *et al*. (2014) are further evaluated in large-scale RCTs.

It is unclear why young people with SoAD experienced poorer outcomes following generic CBT compared with those with other anxiety disorders. One explanation might be that there are specific processes that maintain SoAD, meaning recovery is less likely if these are not specifically addressed in therapy. For example, adult models of SoAD highlight the role of self-focused attention and negative observer-perspective images as key maintaining factors (Hackmann *et al*., 2000; Wong and Rapee, 2016). In CBT for social anxiety in adults, this is typically addressed through a combination of attention training exercises, behavioural experiments aimed at exploring the impact of self-focused attention, and video feedback to correct negative images (Schreiber *et al*., 2015; Warnock-Parkes *et al*., 2017). The inclusion of these treatment components has been shown to be associated with enhanced treatment outcomes (Schreiber *et al*., 2015; Warnock-Parkes *et al*., 2017). Several authors have suggested that the Clark and Wells (1995) model of SoAD, including the role of self-focused attention and negative imagery, is applicable to youth (for a review, see Leigh and Clark, 2018). As generic CBT is unlikely to include procedures to address these disorder-specific maintenance factors, this may explain the poor recovery outcomes observed for youth with primary SoAD. Future research would therefore benefit from exploring this by examining changes in disorder-specific maintenance factors during generic CBT for youth with primary SoAD, to better understand barriers to recovery following this treatment.

It was beyond the scope of this review to consider other predictors of outcome, or the interaction between primary diagnosis and other predictors. This is an important question, especially given findings in one of the included trials (Arendt *et al*., 2016) that youth with primary SoAD were also likely to be older and have higher rates of co-morbid depression. Future research would benefit from further investigating additional factors such as these and their interaction in predicting recovery from anxiety disorders following CBT. It was also not possible to investigate developmental differences in outcome. There is evidence of developmental differences in social anxiety symptoms and presentation between children and adolescents (Rao *et al*., 2007). Moreover, studies of the applicability of the Clark and Wells (1995) model of SoAD and maintenance processes such as safety behaviours, self-focused attention and social cognitions have largely focused on adolescents rather than children (Hodson *et al*., 2008; Leigh and Clark, 2018). It is therefore possible that a different pattern of response to disorder-specific *versus* generic CBT for SoAD will be observed in children compared with adolescents. Future reviews would therefore benefit from comparing outcomes not just between primary diagnoses but also between children and adolescents. Such a review would benefit from also considering outcomes of alternative psychological treatments which have an evidence base for the treatment of SoAD in children, such as social skills interventions (Beidel *et al*., 2000; Beidel *et al*., 2005).

This review has a number of strengths. It included trials of CBT across a range of formats, increasing the generalisability of results. All outcomes were based on validated diagnostic tools, and from RCTs published in peer-reviewed journals. Whilst these criteria led to the exclusion of at least one trial which reported outcomes consistent with the conclusions of this review from a non-RCT design (Crawley *et al*., 2008), overall these criteria were beneficial as they ensured that the review’s conclusions were based on high-quality evidence. This was supported by the findings of the quality assessment, which revealed a generally low level of bias and good quality research evidence.

This review also has several limitations. The included papers represent a minority of trials of CBT for anxiety in children and adolescents, as 67 were excluded from the current review as they did not present sufficient data on recovery rates relative to primary diagnosis. It is possible that this led to bias in our findings, for example it could be possible that trials finding evidence of a difference in recovery rates between diagnoses were more likely to report these data. However, the symmetrical funnel plot provides evidence to suggest that results were not affected by such publication bias. A further limitation is that study screening and selection was primarily completed by only one researcher. Although this process was completed cautiously and followed clearly specified inclusion and exclusion criteria, and ambiguities were discussed and agreed within the research team, a more rigorous approach would have been for double coding of studies by a second researcher. The present review also only focused on recovery outcomes at post-treatment, and therefore does not reflect different recovery outcomes which may have been observed at longer-term follow up. For example, Thirlwall *et al*. (2013, 2017) highlighted that whilst young people with primary GAD showed similar rates of recovery from post-treatment to 6-month follow-up, recovery rates amongst young people with other primary anxiety diagnosis continued to improve between these points. The conclusions of the current review may therefore not be replicated at longer term follow-up points. Additionally, our focus on recovery from primary diagnosis prevented conclusions regarding symptom improvement or recovery from non-primary anxiety diagnoses. It was also not possible to extract data on recovery rates following control conditions. Therefore, it is not possible to establish whether the differences observed in recovery between those with different primary anxiety diagnoses are specific to CBT or are reflective of a more general pattern of recovery across conditions.

These limitations notwithstanding, this review has produced important findings with clear research and clinical implications. It has shown, from a small number of recent trials, that young people with primary SoAD are significantly less likely to be in remission from this diagnosis following generic CBT at post-treatment compared with young people with other primary anxiety disorders. This demonstrates the importance of reporting recovery rates relative to primary diagnosis in all trials of CBT for youth anxiety, and the urgent need for further research to enhance understanding of SoAD in young people in order to improve the efficacy of treatment for children and adolescents with this diagnosis.

## Supplementary material

For supplementary material accompanying this paper visit https://doi.org/10.1017/S135246582000079X. click here to view supplementary material
