# Supplementary material for: Are young people with primary social anxiety disorder less likely to recover following generic CBT compared to young people with other primary anxiety disorders? A systematic review and meta-analysis
Source: Behav Cogn Psychother. 2020 Dec 10;49(3):352–69. doi: 10.1017/S135246582000079X (PMC8293629; doi:10.1017/S135246582000079X)
Supplement: Supplementary file 1 [file S135246582000079Xsup.zip › S135246582000079Xsup001.docx]

Appendix B. Full Electronic Search in Psycinfo Database.

Database: Psycinfo

Date of search: 21.10.2019

1. (Youth OR adolescent OR adolescence OR Child OR CAMHS OR Teenage OR CAMS OR Young people OR Pediatric OR Paediatric).ab.ot.ti.

2. (Anxiety OR Anxiety disorder OR social anxiety disorder OR social phobia OR social anxiety OR Panic disorder OR Specific phobia OR Agoraphobia OR Separation anxiety disorder OR Generalised anxiety disorder OR Generalized anxiety disorder).ab.ot.ti.

3. (Cognitive behavioural therapy OR Cognitive behavioral therapy OR Cognitive behaviour therapy OR Cognitive behavior therapy OR Cognitive therapy OR CBT).ab.ot.ti.

4. Limit 1 to (English language and yr=”1990-2019”)

5. Limit 2 to (English language and yr=”1990-2019”)

6. Limit 3 to (English language and yr=”1990-2019”)

7. 1 and 2 and 3 and 4 and 5 and 6
