# Supplementary figures and images for: Are young people with primary social anxiety disorder less likely to recover following generic CBT compared to young people with other primary anxiety disorders? A systematic review and meta-analysis
Source: Behav Cogn Psychother. 2020 Dec 10;49(3):352–69. doi: 10.1017/S135246582000079X (PMC8293629; doi:10.1017/S135246582000079X)

Appendix C. Funnel plot of 6 trials included in the meta-analysis.


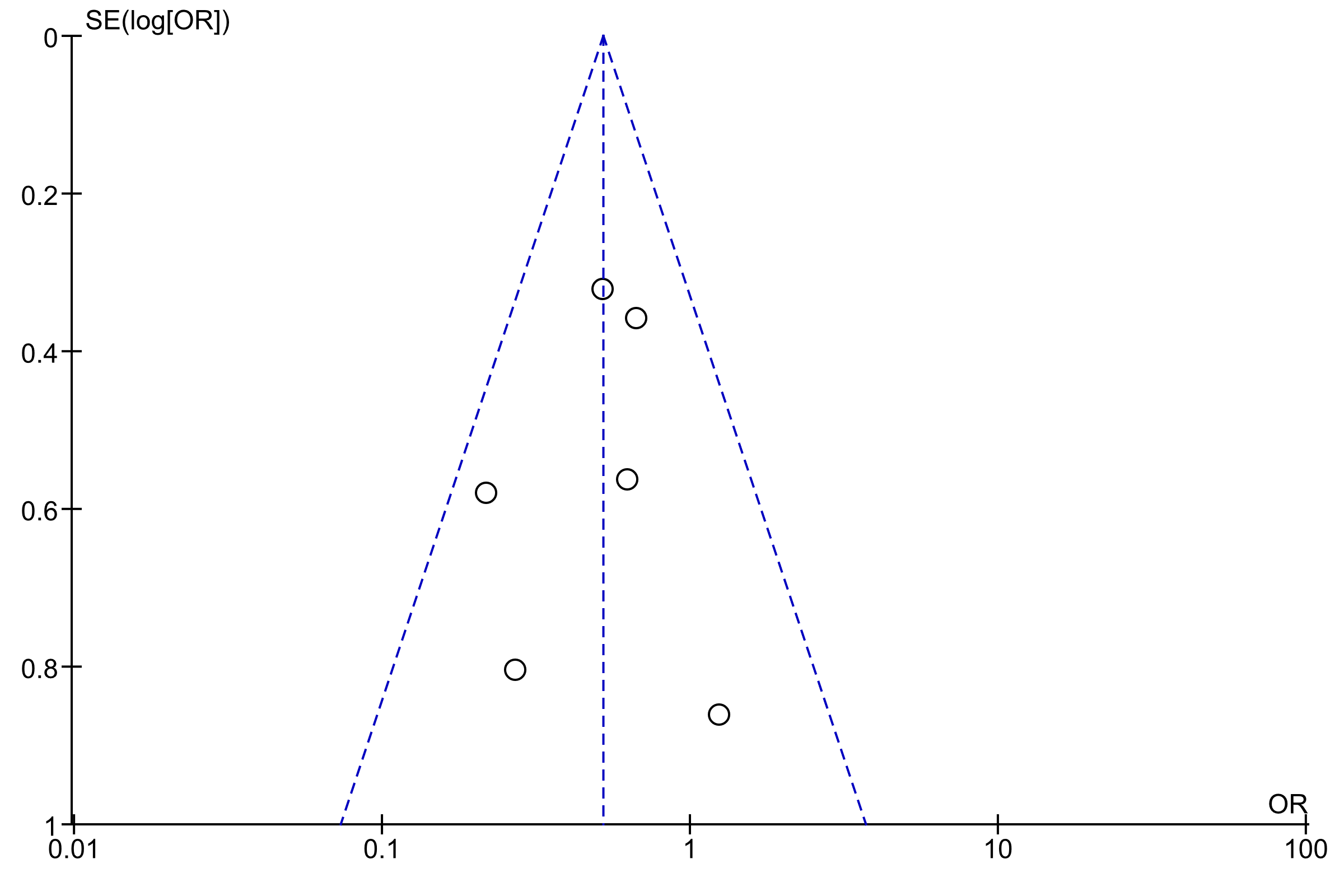

Supplement: Supplementary file 1 [file S135246582000079Xsup.zip › S135246582000079Xsup003.docx]
